# Successful whole lung lavage in pulmonary alveolar proteinosis secondary to lysinuric protein intolerance: a case report

**DOI:** 10.1186/1750-1172-2-14

**Published:** 2007-03-26

**Authors:** Michele Ceruti, Giuseppe Rodi, Giulia M Stella, Andrea Adami, Antonia Bolongaro, Aldo Baritussio, Ernesto Pozzi, Maurizio Luisetti

**Affiliations:** 1 Clinica Malattie Apparato Respiratorio, Fondazione IRCCS Policlinico San Matteo, Università di Pavia, Italy; 2 Servizio di Anestesia e Rianimazione I, Fondazione IRCCS Policlinico San Matteo, Università di Pavia, Italy; 3 Dipartimento di Pediatria, Ospedale San Carlo Borromeo, Milano, Italy; 4 Dipartimento di Scienze Mediche e Chirurgiche, Clinica Medica I, Università di Padova, Italy

## Abstract

**Background:**

Pulmonary alveolar proteinosis (PAP) is a rare disease characterised by accumulation of lipoproteinaceous material within alveoli, occurring in three clinically distinct forms: congenital, acquired and secondary. Among the latter, lysinuric protein intolerance (LPI) is a rare genetic disorder caused by defective transport of cationic amino acids. Whole Lung Lavage (WLL) is currently the gold standard therapy for severe cases of PAP.

**Case presentation:**

We describe the case of an Italian boy affected by LPI who, by the age of 10, developed digital clubbing and, by the age of 16, a mild restrictive functional impairment associated with a high-resolution computed tomography (HRCT) pattern consistent with pulmonary alveolar proteinosis. After careful assessment, he underwent WLL.

**Conclusion:**

Two years after WLL, the patient has no clinical, radiological or functional evidence of pulmonary disease recurrence, thus suggesting that WLL may be helpful in the treatment of PAP secondary to LPI.

## Background : pulmonary involvement in LPI

Pulmonary alveolar proteinosis (PAP) is a rare disease characterized by the accumulation of a lipoproteinaceous, eosinophilic, periodic acid-Schiff (PAS) positive material within alveoli [1]. The disease was first described in 1958 [2] and up to 2002 about 410 cases have been reported in the literature [3]. The clinical course of the disease is variable, ranging from severe respiratory failure to spontaneous remission. PAP occurs in three distinct forms: congenital, acquired and secondary. The congenital form derives from mutations in the genes encoding surfactant protein (SP) B or C or the **β**c chain of the receptor for the granulocyte-macrophage colonystimulating factor (GM-CSF). Acquired (or idiopathic) PAP is the most common form, occurring in previously healthy adults and is nowadays considered an autoimmune disease in which circulating anti GM-CSF neutralizing autoantibodies play a significant role [4]. Secondary PAP is associated with conditions that lead to functional impairment or reduced numbers of alveolar macrophages, such as some hematologic malignancies, inhalation of inorganic dusts or toxic fumes, pharmacologic immunosuppression, certain infections and, as in the hereby described case, Lysinuric Protein Intolerance (LPI).

LPI (MIM# 222700) is an inherited autosomal recessive hyperdibasic aminoaciduria caused by defective cationic amino acid (CAA; L-arginine, L-lysine, L-ornithine) transport, normally exerted by the y+L system, at the basolateral membrane of epithelial cells in the intestine and kidney [5]. Clinical findings in LPI patients include: vomiting, diarrhea, failure to thrive, hepatosplenomegaly, bone marrow abnormalities, osteoporosis, episodes of coma, mental retardation, altered immune response, chronic renal disease and lung involvement, mainly PAP [6]. LPI is caused by mutations of the solute carrier family 7A member 7 (*SLC7A7*) gene [7,8]. The *SLC7A7 *(MIM# 603593) gene encodes the y+LAT-1 protein which is the light chain subunit of a member of the heterodimeric amino acid transporters (HATs) family.

LPI is a very rare disease which was first described in 1965 in two infant Finnish siblings [9]. The majority of cases reported in the literature concern patients from Finland, where the disease prevalence is about 1 in 60,000 [10].

In 1993, Parto et al [10] reported data from 31 Finnish patients emphasising that children with LPI are highly predisposed to develop PAP and that most adult LPI patients, even though asymptomatic, show radiologic signs of interstitial lung disease.

In 1993, Kerem et al [11] reported the case of a 11-year-old Arabic girl affected by LPI with chronic interstitial lung disease and pulmonary cholesterol granulomas in lung biopsy (no evidence of PAP was found). The authors observed that lung involvement in LPI patients may be either acute (and life threatening) or chronic (as in the case reported).

In 1992, Fisher et al [12] described the different pathologic patterns of endogenous lipid deposition within the lung, as found in eight pediatric patients with varying primary disease (one with LPI): endogenous lipoid pneumonia (ELP), PAP, and pulmonary interstitial and intra-alveolar cholesterol granulomas (PICG) were described, often coexisting in the same patient. The patient affected by LPI had two subsequent lung biopsies revealing signs consistent with ELP, but at autopsy PAP was the most striking pattern.

In 1993, Di Rocco et al [13] reported data from three Italian LPI patients with coexisting interstitial lung disease. The nature of lung involvement was not specified for any of the patients.

In 1994, Parto et al [14] reported again on the four pediatric patients already mentioned [10] who died in respiratory failure, providing more detailed histologic descriptions. They concluded that PAP and cholesterol granulomas, the two main histologic findings in the lung specimens of patients with LPI, could represent different stages of the same pulmonary reaction, and hypothesized that the increased amount of CAA in the alveolar lining in LPI might predispose the patient for PAP development. In another paper, the same authors [15] reported on the ultrastructural analysis of lung specimens from these patients (histologic evidence of PAP in three and PICG in one). They found that pulmonary macrophages from these LPI patients with lung involvement contained significantly more multilamellar structures than did control cells and showed electron-dense material containing an excess of iron. They stated that the increased concentrations of CAA in the alveolar lining might interfere with phospholipid metabolism (and thus predispose for PAP [14]).

In 1995, Parenti et al [16] performed a retrospective analysis in a cohort of nine patients from southern Italy: they reported that respiratory involvement (evident in chest radiographs or HRCT scans) was present in five cases, with histologically proven PAP in one patient, who died at the age of 11 years.

In 1996, McManus et al [17] reported the necropsy findings from a 21 year old female with LPI, who died from a rapidly progressive multiple organ failure. The lungs showed histological PAP.

Since pulmonary involvement represents one of the major causes of an impaired clinical course and fatal outcome in LPI, early diagnosis and subsequent monitoring of such manifestations should be mandatory. In 1996 Santamaria et al [18] reported the radiological and functional data of an Italian cohort of nine patients, already described in [16]. HRCT and perfusion/ventilation scintigraphy appeared to be the most sensitive methods to detect lung involvement in LPI, even in patients without clinical and functional impairment.

In 2004, Santamaria et al [19] reported on the case of an Italian boy with LPI and PAP. Whole Lung Lavage (WLL) was performed on two separate occasions, without clinical improvement. The patient, at the age of 3 years, underwent a hearth-lung transplantation. Eighteen months later the patient developed a severe Epstein Barr Virus pneumonia and HRCT scans and transbronchial lung biopsy showed findings consistent with PAP recurrence in the donor pulmonary graft. On two separate occasions the patient was retreated with WLL, without any improvement. The patient died 26 months after transplantation. Before and after lung transplantation an attempt with subcutaneous GM-CSF therapy (6 **μ**g/kg per day) was made and discontinued after respectively 8 and 6 days due to the emergence of side effects and excessive leucocytosis.

The number of patients affected by LPI described in the above summary is less than 50 and the number of patients with a proven diagnosis of PAP is 7. The estimated number of LPI cases diagnosed worldwide, as of 1994, was about 80 [15], and according to Simell [6] the number may rise to over 100. Based on our search of the literature, the above reported [19] is the only, unsuccessful, case of PAP secondary to LPI treated with WLL. Here we report on the case of a boy affected by PAP secondary to LPI, successfully treated with WLL, with sustained and long standing remission.

## Case presentation

The proband was an Italian boy, currently aged 17. At eight months of age he was diagnosed LPI, with coexisting glucose-6-phosphate dehydrogenase (G6PD) deficiency and VII clotting factor deficiency. Interestingly his clinical case had already beeen reported by other authors [20], but at that time pulmonary involvement was not indicated.

### Development of lung involvement and diagnosis of PAP

Since 1999, the patient has been followed for the development of a mild and stable restrictive functional impairment, associated in the following years with development of digital clubbing; the patient did not complain for the presence of dyspnoea. On march 2004, the patient reported atypical abdominal and thoracic pain. A computed tomography (CT) of the abdomen was performed and resulted negative except for the images including part of the lower pulmonary lobes, showing areas of ground glass attenuation with interlobular septa thickening, a pattern strongly suggesting PAP. Routine laboratory tests were negative. The restrictive ventilatory defect had worsened but respiratory failure was not present; the patient started reporting episodes of dyspnoea. Steroid therapy (prednisone 1 mg/kg/die) was started with close surveillance for possible side effects on bone metabolism. Considering that PAP in LPI pediatric patients is a serious and possibly fatal complication, despite the absence of respiratory failure the patient was referred to our centre in Pavia, where WLL is standard procedure for patients with PAP [21]. In June 2004, the patient was admitted to the Pavia Hospital. He still presented with annoying thoracic pain; at physical examination of the chest fine crackles were present; pulmonary function testing showed a moderate restrictive ventilatory defect (Table 1); respiratory failure was not present (in room air PaO_2 _10.3 kPa, PaCO_2 _5.3 kPa); routine laboratory investigations showed mild anemia, thrombocytopenia, marked increase in lactate dehydrogenase; chest HRCT was consistent with the PAP diagnosis; the bronchoalveolar lavage fluid (BALf) had a milky appearance and was rich in foamy macrophages filled with cholesterol crystals in a diffuse background of granular eosinophilic amorphous material. The diagnosis of PAP was confirmed based on the HRCT pattern and BALf characteristics.

**Table 1:** Pulmonary function testing before and 9 months after WLL

	BEFORE WLL		9 MONTHS AFTER WLL	
	
	Value	% predicted	Value	% predicted
VC	1,09 L	44	1,37 L	51
FRC	0,94 L	70	1,29 L	86
RV	0,49 L	79	0,69 L	103
TLC	1,58 L	55	2,06 L	65
FEV_1_	0,94 L/s	42	1,06 L/s	44
FEV_1_/VC	86,24%	102	77,37%	92
RV/TLC	31,01%	151	33,5%	163

Pulmonary function parameters, measured one day before and about 9 months after WLL; mild restrictive impairment is present, with a slight improvement after the procedure. VC, vital capacity; FRC, functional residual capacity; RV, residual volume; TLC, total lung capacity; FEV1, forced expiratory volume in 1 second.

### WLL treatment

**Figure 1:**
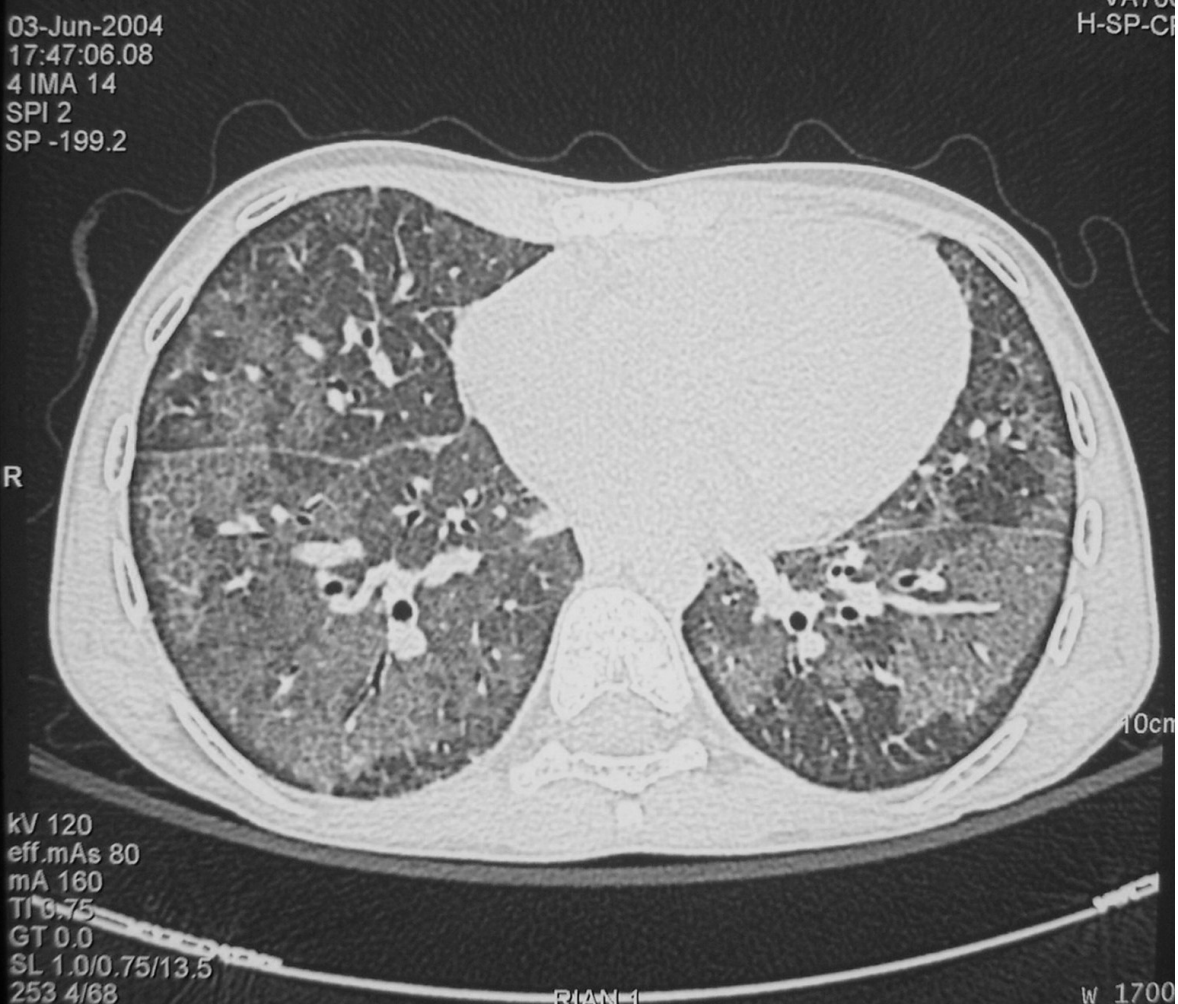
**Chest HRCT performed 1 day before WLL**. Areas of ground glass attenuation with concomitant interlobular septa thickening are evident, alternated with areas of normal lung (crazy paving pattern).

On June 4^th^, 2004, the patient was treated with WLL in the intensive care unit. A chest HRCT was performed the day before the lavage (Figure 1). Before starting the procedure, the patient underwent a plasma transfusion in order to correct clotting deficiency. During WLL a nurse assisted the chest wall percussions, which require particular care in patients with osteoporosis. The lungs were sequentially lavaged, adopting the well established technique used at our centre, already described in detail [21,22]. The WLL was successufully performed with satisfactory recovery of outflow fluid (8 L for the left lung, 9 L for the right); no complications during or after WLL were observed. The analysis of the outflow fluid confirmed that phospholipids, total protein and SP-A concentration were remarkably alterated (Table 2).

**Table 2:** Proteins, phospholipids and SP-A concentrations in WLL outflow fluid.

	Total proteins (μg/mL)	Phospholipids (μg/mL)	SP-A (μg/mL)
Right lung	8,500	150	202
Left lung	6,500	80	41
Controls (mean ± SD)	171 ± 74	46 ± 27	1 ± 1

Concentrations of total proteins, phospholipids and SP-A were measured in the outflow fluid after lavage of both lungs and were found to be markedly increased with respect to control levels.

### Two year follow-up

**Figure 2:**
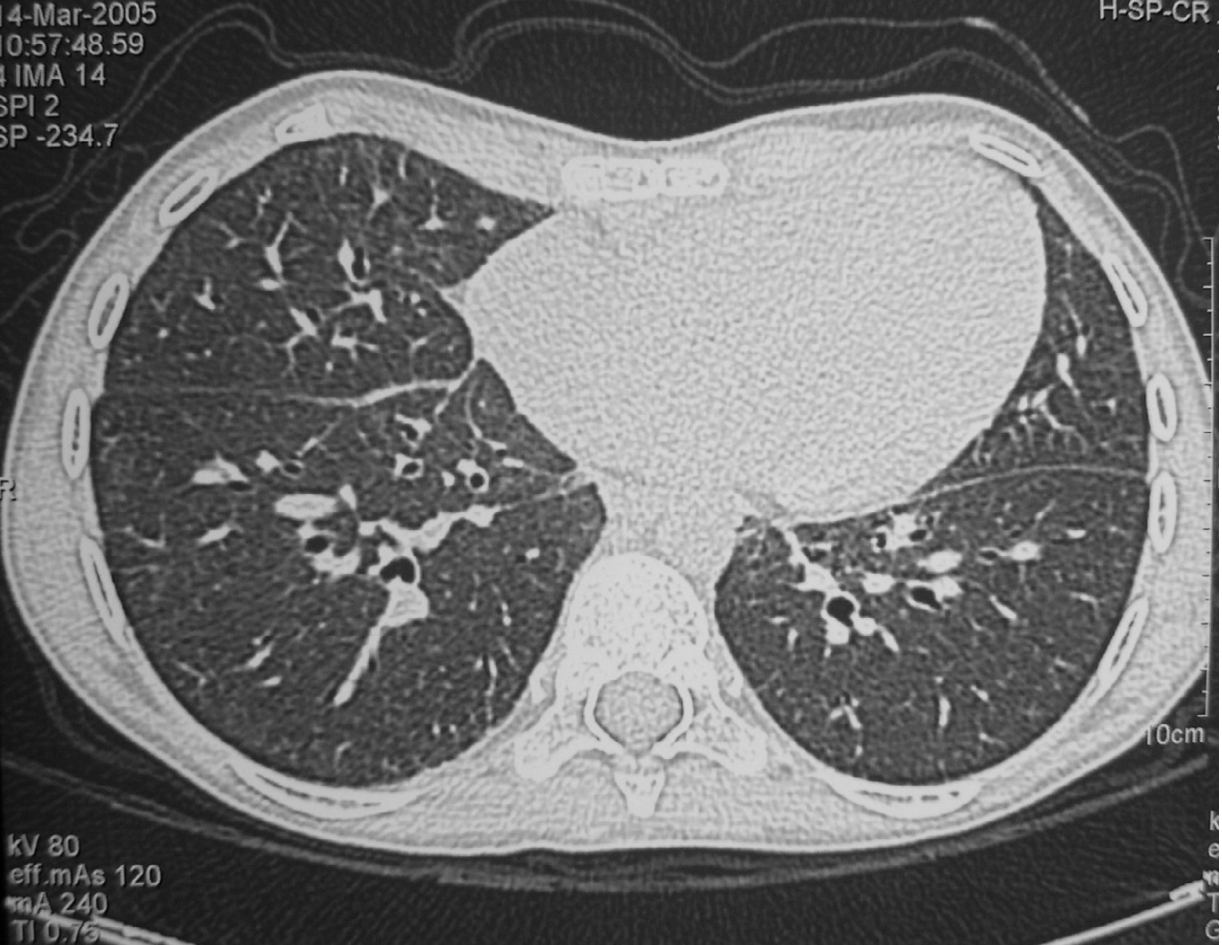
**Chest HRCT performed 10 months after WLL**. Areas of ground glass attenuation are no longer visible; only minimal interlobular septa thickening is still evident.

In the following two years the patient has maintained a satisfactory respiratory status, assessed at regular visits in the pediatric department. In March 2005, about 9 months after WLL, he was re-evaluated with complete functional tests and chest HRCT. Pulmonary function testing showed a little improvement, as compared with the pre-WLL test (Table 1); arterial oxygen saturation, assessed with a pulse-oximeter, was in the normal range (97 %); exercise testing (modified Bruce protocol) showed normal exercise tolerance, and was conducted up to stage 4, with a total distance covered before exercise interruption of 1172 m (the test was not performed before WLL because of chest pain reported by the patient). Chest HRCT (Figure 2), in comparison with the exam performed immediately before WLL, showed only minimal, isolated, interlobular septa thickening in the lower portions of the lower lobes; parenchymal density was diffusely increased (650–750 Hounsfield Units) in respect to normal values, even in absence of ground glass attenuation, but consistent with the mild functional restrictive impairment. In this setting, anti GM-CSF autoantibodies were tested and resulted negative (less than 3 **μ**g/mL).

To date, the patient is in good general condition and has not reported any recurrence of respiratory symptoms. Regular follow-up visits are still performed in the pediatric department, with periodic respiratory function tests and chest X-ray.

## Conclusion

To the best of our knowledge, this is the first reported case of PAP secondary to LPI succesfully treated with WLL. One case has already been reported [19] where WLL was performed on two separate occasions without any improvement. However, lack of efficacy in this case may be attributed to post lung transplant complications. It has also been observed that WLL is the only effective treatment for severe cases of PAP in children [23]. The efficacy of the procedure in adults is well established [21] and successful use of multiple total lung lavages in infants and paediatric patients with PAP has already been reported [24]. Taking into account the fact that lung involvement is a serious and possibly fatal complication in LPI, we decided to perform a WLL in our patient, even in the absence of respiratory failure. This decision was also made in order to prevent further deterioration of his respiratory condition that would have led to an urgent WLL.

It has been previously observed that there are no data concerning anti GM-CSF autoantibodies in PAP secondary to LPI [19]. Determination of anti GM-CSF auto-antibodies in 15 children and 3 neonates affected by PAP revealed that in most of the children and all of the neonates the anti GM-CSF titres were not significantly increased [25]. In addition, mutations of surfactant protein B gene and other known external or syndromic causes of PAP, including LPI, were ruled out in this cohort of paediatric patients. Therefore, this appears to be the first reported titre of anti-GM-CSF autoantibodies in a patient with PAP secondary to LPI. The determined level (less than 3 **μ**g/mL) was negative for the presence of such antibodies as expected and is consistent with the secondary form of PAP, in which the anti-GM-CSF auto-antibodies do not play a role.

Pathogenesis of lung involvement in LPI remains enigmatic. Various hypotheses have been proposed. A possible explanation is that increased concentration of CAA in the alveolar lining might interfere with cell membrane and surfactant turnover and thus predispose the patient to the development of ELP, PICG or PAP [14,15], which represent the forms of endogenous lipid deposition within the lung that by some are considered to be different stages of lung involvement in LPI. An alternative hypotheses is that PAP secondary to LPI might be considered a disorder of bone marrow derived monocytes. It is well known that bone marrow abnormalities may be found in LPI patients (most frequently erythroblastophagocytosis) and it has been proposed that primarily altered monocytes may differentiate into dysfunctional alveolar macrophages [19]. Based on this hypothesis, bone marrow tranplantation could be beneficial in LPI complicated by PAP [19]. Recently, it has been found that nitrate levels are increased in the plasma of patients with LPI: the Authors hypothesized that the increased nitric oxide production could play a role in the pathogenesis of poorly understood conditions associated with LPI, such as PAP [26].

Uncertainities regarding the pathogenesis of the disease have severely limited the aetiologic treatment of PAP in LPI. There is no evidence on the effectiveness of GM-CSF replacement for this condition: furthermore, it does not seem that GM-CSF signalling is impaired in PAP complicating LPI, thus suggesting a lack of rationale for substitutive treatment. Bone marrow transplantation is an intriguing therapeutic option but, to date, there are no reported cases on its efficacy in PAP secondary to LPI and a better understanding of bone marrow abnormalities in LPI is needed. WLL, which provides removal of the accumulated material from the alveolar spaces, is thus an effective and safe procedure [23,24]. Our case demonstrates that, since PAP in LPI is a life-threatening complication, WLL should be accurately evaluated in all patients with LPI and severe lung involvement. Further studies are needed to ascertain if WLL is a therapeutic option in patients who have mild respiratory impairment, or show a trend for progressive deterioration of lung function.

## Competing interests

The author(s) declare that they have no competing interests.

## Authors' contributions

MC and GMS performed the literature search and drafted the manuscript, GR and ABo planned and performed the WLL, AA dealt with the patient, AB performed the biochemical analysis of the BALf, EP partecipated in the design of the study, ML partecipated in the desing of the study and supervised the manuscript
